# Radical nephrectomy combined with removal of tumor thrombus from inferior vena cava under real-time monitoring with transesophageal echocardiography: A case report

**DOI:** 10.1097/MD.0000000000019392

**Published:** 2020-03-13

**Authors:** Yaozhu Wang, Xu Wang, Yuan Chang

**Affiliations:** a Department of Anesthesiology, Shandong Provincial ENT Hospital, Shandong Provincial ENT Hospital affiliated to Shandong University; b Department of Anesthesiology, Shandong Provincial Hospital Affiliated to Shandong University. Jinan 250021, Shandong Province, China.

**Keywords:** anesthesia, inferior vena cava, renal carcinoma, right atrium, transesophageal echocardiography, tumor thrombus

## Abstract

**Rationale:**

Renal carcinoma is a common malignant tumor of the urinary system, 4%–10% of which are complicated with tumor thrombi in the renal vein and the inferior vena cava; in about 1% of patients, the inferior vena cava tumor thrombus invades the right atrium. Surgery is the treatment of choice. Real-time monitoring with transesophageal echocardiography (TEE) has been widely used in various operations, including cardiac and non-cardiac operations for congenital heart diseases, coronary diseases, vascular heart diseases, and aorta diseases, etc. In this article, a case of a patient with right renal carcinoma complicated with an inferior vena cava tumor thrombus is reported.

**Patient concerns:**

A 52-year-old man who was admitted to our hospital for lumbar pain lasting for one month.

**Diagnosis:**

Right renal carcinoma complicated with an inferior vena cava tumor thrombus.

**Interventions:**

Radical nephrectomy of the renal carcinoma and removal of an inferior vena cava tumor thrombus under real-time monitoring with TEE were performed.

**Outcomes:**

Radical nephrectomy was successfully performed within 5 minutes after the inferior vena cava was clamped, and then the inferior vena cava tumor thrombus was removed. On the second day after the operation, the patient's conditions improved; his consciousness was clear; he was transferred to a general ward. On the third day after the operation, the patient was able to get out of bed and was discharged on the sixth day after the operation.

**Lessons:**

Real-time monitoring with TEE played an important role in many aspects in the radical nephrectomy of the renal carcinoma and removal of the inferior vena cava tumor thrombus.

## 1 Introduction

Renal carcinoma is a common malignant tumor of the urinary system; 4% to 10% of patients are complicated with tumor thrombus in the renal vein and the inferior vena cava;^[1]^ in ∼1% of patients, the inferior vena cava tumor thrombus invades the right atrium.^[2]^ Surgery is the treatment of choice. Real-time monitoring with transesophageal echocardiography (TEE) has been widely used in various operations, including cardiac and noncardiac operations for congenital heart diseases, coronary diseases, vascular heart diseases, and aorta diseases, etc.^[3]^ A case of a patient with right renal carcinoma complicated with an inferior vena cava tumor thrombus is reported in this article.

Written consent was obtained from the patient for publication of this case report.

## 2 Case report

The patient, male, 52 years old, 78 kg, was admitted to our hospital due to low back pain for 1 month and aggravation for 2 weeks. Abdominal ultrasonography and computed tomography (CT) showed a right kidney tumor (with the maximum section of about 6.3 × 11.3 cm) with a tumor thrombus in the right renal vein and inferior vena cava (with the maximum section of ∼3.4 × 2.9 cm), with the upper edge of the thrombus at the right atrium entrance. Preliminary diagnosis: right renal carcinoma with an inferior vena cava tumor thrombus. Mayo grading was Level IV.^[4]^ Radical nephrectomy of the renal carcinoma and removal of the inferior vena cava tumor thrombus were intended to be performed under general anesthesia. The American Society of Anesthesiologists (ASA) grading of the renal carcinoma was Level III.

**Figure 1:**
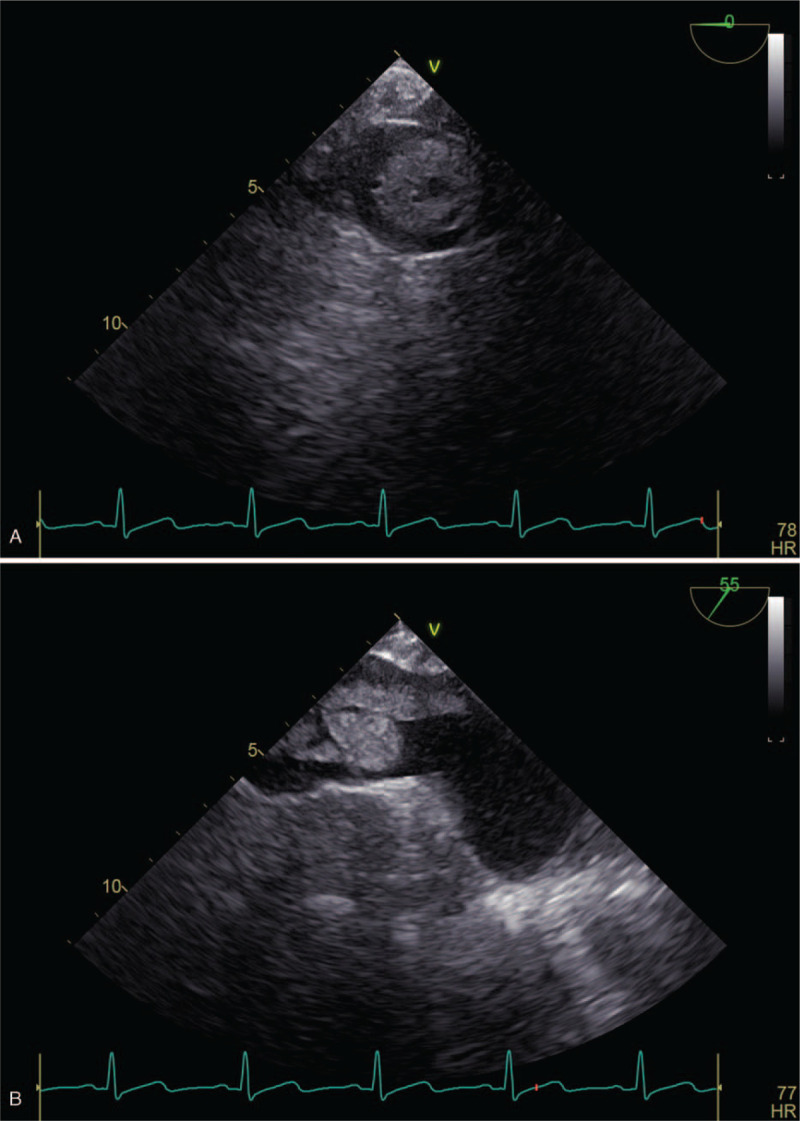
(A) TEE of tumor thrombus. (B) TEE of tumor thrombus. TEE = transesophageal echocardiography.

After the patient entered the operation room, venous access was established. Electrocardiogram (ECG), heart rate (HR), and oxygen saturation (SpO_2_) were routinely monitored, and radial artery puncture and cannulation were performed with the adult 7# dual-lumen catheter kit. Anesthesia induction: midazolam 2 mg, sufentanil 15 μg, etomidate 18 mg and cis-atracurium 20 mg were intravenously injected. Anesthesia maintenance: propofol 5 mg kg^−1^ h^−1^ was intravenously infused, and 2% sevoflurane was inhaled. Examination showed HR 63 times/min, blood pressure 115/65 mm Hg, SpO_2_ 100%, central venous pressure (CVP) 15 mm Hg, and partial pressure of carbon dioxide (PCO_2_) 47 mm Hg. When anesthesia was successfully achieved, a transesophageal ultrasound probe was inserted into the esophagus through the patient's oropharynx; the tumor thrombus was confirmed as a floating embolus with the probe (Fig. 1A). The upper edge of the tumor thrombus was ∼1.9 cm from the opening of the right atrium (Fig. 1B). It was decided that surgical procedures would be performed under real-time monitoring of the tumor thrombus with TEE and without cardiopulmonary bypass being established.

**Figure 2:**
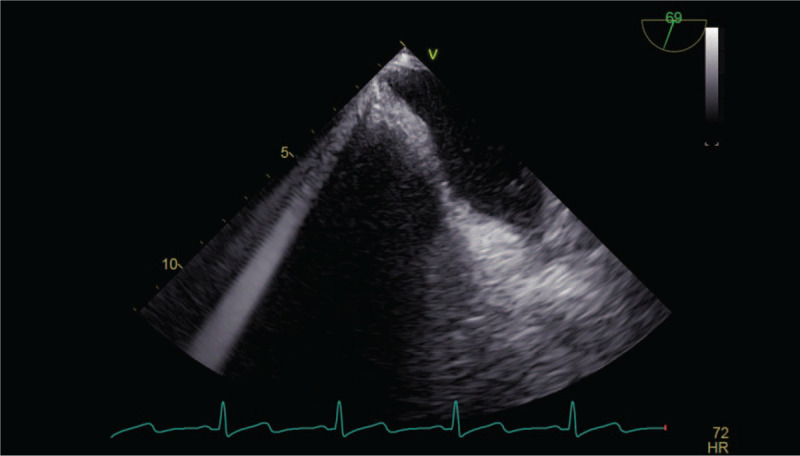
TEE of clamping of the inferior vena cava. TEE = transesophageal echocardiography.

**Figure 3:**
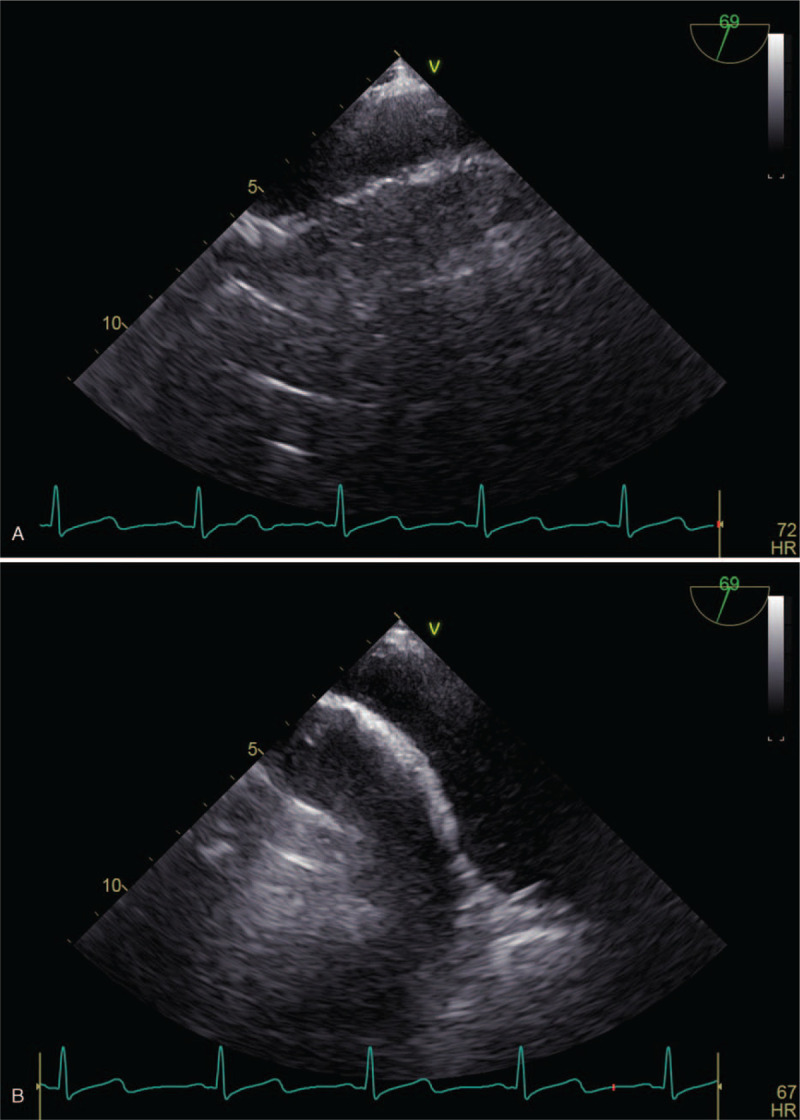
(A) TEE after the tumor thrombus was removed. (B) TEE after the tumor thrombus was removed. TEE = transesophageal echocardiography.

TEE during the dissection process of renal carcinoma showed that the end of the tumor thrombus entered the right atrium twice, which prompted the surgeon to withdraw the tumor thrombus from the right atrial opening in time. About 105 minutes after the start of the operation, the inferior vena cava at the entrance to the atrium was blocked under real-time monitoring with TEE, and the inferior vena cava was clamped with the end of the tumor thrombus being avoided accurately. After successful clamping of the inferior vena cava (Fig. 2), TEE showed a significant reduction in blood flow to the heart; physical examination showed HR 65 times/min, blood pressure 85/63 mm Hg, SpO_2_ 97%, and CVP 3 mm Hg. Blood pressure was maintained with intermittent intravenous injection of phenylephrine during the clamping. The renal carcinoma was successfully removed within 5 minutes after the inferior vena cava was clamped, and the inferior vena cava tumor thrombus was removed (Fig. 3A and B). After recovery of blood flow, physical examination showed HR 69 times/min, blood pressure 140/80 mm Hg, SpO_2_ 97%, CVP 12 mm Hg, and PCO_2_ 44 mm Hg. The intraoperative bleeding volume was about 200 mL. After surgery, the patient was admitted to the intensive care unit (ICU) with a trachea cannula inserted. According to the follow-up, the trachea cannula was removed 3 hours after admission to the ICU. Physical examination showed HR 78 times/min, blood pressure 135/75 mm Hg, and SpO_2_ 97%. On the second day after the operation, the patient's conditions improved, his consciousness was clear, and he was transferred to a general ward. On the third day after the operation, the patient was able to get out of bed and was discharged on the sixth day after the operation. No recurrence and the clinical symptoms were observed during the 8 months’ follow-up.

## 3 Discussion

According to the anatomical locations of the renal carcinoma and the inferior vena cava tumor thrombus, the tumors are graded by the Mayo Clinic into 5 levels. Different Mayo levels are associated with different surgical complexities, blood loss, transfusion rates, and perioperative complications.^[4]^ According to the 5-level classification mentioned above, in this case it was Mayo Level IV, that is, the tumor thrombus invaded the inferior vena cava above the diaphragm, and it was necessary to remove the tumor thrombus with cardiopulmonary bypass (CPB).^[5]^ However, with CPB, the risks of complications such as CPB-related nervous system damage, ischemic injury of parenchymatous organ, and postoperative coagulopathy also increase;^[6]^ intense hemodynamic fluctuations often occur during the perioperative period; even serious events such as ventricular inflow–outflow obstruction, pulmonary embolism (PE), and cardiac arrest may occur, with a mortality rate of 6% to 9%.^[7]^ The American Ultrasound Association has pointed out^[8]^ that TEE has an important application value in resection of renal carcinoma with inferior vena cava tumor thrombus.

In this case, TEE real-time monitoring played an important role in many aspects, including: Confirming the diagnosis and determining the anesthesia and surgical plan. With perioperative TEE, we could accurately locate the inferior vena cava tumor thrombus in real time, and assess the size, length, and nature (floating, invasive) of the tumor thrombus and the invaded blood vessels, thereby optimizing anesthesia and surgical decision making. In this case, it was confirmed with TEE monitoring that the tumor thrombus was floating near the proximal right atrium opening (1.9 cm), therefore, laparotomy was performed under general anesthesia without CPB being established. Real-time monitoring to prevent the tumor thrombus from falling off. PE is the main cause of death in the resection process of renal carcinoma complicated with an inferior vena cava tumor thrombus.^[9,10]^ Perioperative PE has occurred in ∼6% of patients with an inferior vena cava tumor thrombus, with the mortality rate as high as 60% to 75%.^[11]^ PE tends to occur in the dissection process of the kidney and the inferior vena cava and clamping of the inferior vena cava. With TEE monitoring, the end of the tumor thrombus can be observed in real time and its movement can be monitored. In this case, TEE prompted the surgeon that the end of the tumor thrombus extended into the right atrium during the dissection process of the renal carcinoma, avoiding the “cutting” of the end of the tumor thrombus by the tricuspid valve. Providing guidance and precise blocking. The surgical procedures can be performed under direct vision with TEE, thus it can prevent the tumor thrombus from falling due to insufficient blocking range and excessive dissection.^[12]^ In this case, with TEE real-time monitoring, a pair of vascular occlusion forceps was placed in the tumor thrombus-free length 1.9 cm from the right atrial opening, avoiding the clamping of the tumor thrombus or the atrial coronary sinus, and it could be monitored whether there was cancer thrombus or air embolism. Continuous monitoring of hemodynamics. In the resection process of renal carcinoma complicated with an inferior vena cava tumor thrombus, if there are sudden hemodynamic fluctuations, TEE can enable rapid diagnosis and guide the corresponding treatment measures to quickly restore stable circulation; with the monitoring data obtained with TEE, perioperative circulation management is sublimated from experience to dialectical decisions, which makes the hemodynamic management more refined.^[13]^ Reducing trauma and accelerating recovery. In recent years, it has been reported^[14,15]^ that with TEE monitoring, the transabdominal transdiaphragmatic approach is used to treat the tumor thrombus above the diaphragm. A tumor thrombus above the diaphragm may move to below the diaphragm through dissection of the central tendon of the diaphragm or incision of the diaphragm, thus the tumor thrombus can be successfully removed without thoracic surgery and CPB, and with significantly reduced complications. As a monitoring tool, TEE can guide the implementation of perioperative goal-directed therapy (GDT), so as to accelerate postoperative functional recovery and shorten hospitalization time, which meets the fundamental requirements of the enhanced recovery after surgery (ERAS) concept.

The clinical application of radical nephrectomy combined with removal of the inferior vena cava tumor thrombus under TEE real-time monitoring also has some objective limitations, which consist mainly of the need of specialized ultrasound equipments and professionally trained researchers. In addition, transesophageal echocardiography is a semi-invasive test and is contraindicated or should be used with caution in patients with acute myocardial infarction, heart failure, serious arrhythmia, esophageal stricture or varicose veins, and patients with mental disorders who cannot cooperate.

## Acknowledgments

The authors would like to thank Dr Gongming Wang, Department of Anesthesiology, Shandong Provincial Hospital Affiliated to Shandong University, Dr Runjia Wang, Department of Anesthesiology, Shandong Provincial Hospital Affiliated to Shandong University, Dr Lifeng Wang, Department of Anesthesiology, Shandong Provincial Western Hospital and Dr Xinping Wen, Department of Orthopedics, Qingdao University affiliated Hospital for their helpful advice.

## Author contributions

**Conceptualization:** Yaozhu Wang, Yuan Chang.

**Data curation:** Yaozhu Wang, Xu Wang, Yuan Chang.

**Formal analysis:** Yaozhu Wang, Yuan Chang.

**Supervision:** Yaozhu Wang, Xu Wang, Yuan Chang.

**Writing – Original Draft:** Yaozhu Wang.

**Writing – Review & Editing:** Yuan Chang.
